# Postmortem Aqueous Humor Analysis in Pigs as an Index of Antemortem Serum Biochemistry Profile and Diagnostic Aid in Animal Welfare

**DOI:** 10.3390/ani16091358

**Published:** 2026-04-29

**Authors:** Željko Mihaljević, Ksenija Šandor, Šimun Naletilić, Zdravka Vidić, Iva Kilvain, Marica Lolić

**Affiliations:** 1 Laboratory for Pathology, Department for Pathological Morphology, Croatian Veterinary Institute, Savska Cesta 143, 10000 Zagreb, Croatia; miha@veinst.hr; 2 Laboratory for Analysis of the VMPs, Department of Veterinary Public Health, Croatian Veterinary Institute, Savska Cesta 143, 10000 Zagreb, Croatia; 3 Laboratory for Diagnostics, Branch Laboratories in Split, Croatian Veterinary Institute, Poljička Cesta 33, 21000 Split, Croatia; z.romac.vzs@veinst.hr; 4 Laboratory for Diagnostics, Branch Laboratories in Rijeka, Croatian Veterinary Institute, Podmurvice 29, 51000 Rijeka, Croatia; kilvain.vzr@veinst.hr; 5 Laboratory for Diagnostics, Branch Laboratories in Vinkovci, Croatian Veterinary Institute, Ul. Josipa Kozarca 24, 32100 Vinkovci, Croatia; lolic@veinst.hr

**Keywords:** aqueous humor, biochemical analysis, blood, ventilation system failure, forensic, pig

## Abstract

The present study aimed to assess whether postmortem analysis of aqueous humor in pigs can be used to estimate antemortem serum biochemical values. The experimental design used a control group to establish regression equations linking postmortem aqueous humor to antemortem serum biochemical values. These models enabled reconstruction of the physiological status in decomposed forensic cases associated with heatstroke and hypoxia in pigs that died following a ventilation system failure on a commercial farm, and assessment of physiological distress, cause of death, and potential intentional animal abuse. Concentrations of albumin (ALB), alkaline phosphatase (ALP), alanine aminotransferase (ALT), amylase (AMY), total bilirubin (TBIL), urea nitrogen (UN), creatinine (CRE), calcium (Ca), phosphate (PHOS), sodium (Na), potassium (K), glucose (GLU) and total protein (TP) were measured in aqueous humor and compared with serum samples obtained after slaughter of 30 pigs. Biochemical analyses were performed using a chemistry analyzer with commercial reagent rotors designed. Strong correlations were observed for Na, K and CRE concentrations and for ALT and UN activities between aqueous humor and serum, while TP, ALB, AMY, TBIL and Ca showed weaker associations. Notably, CRE and UN showed strong postmortem correlations with serum values in pigs, consistent with findings in cats and other species, highlighting their reliability as indicators of renal function. Electrolyte concentrations, particularly K and Na, followed consistent and well-recognized patterns described in both human and veterinary forensic studies, with K levels in pigs comparable to those observed in other domestic animals. The results indicate that postmortem aqueous humor analysis of CRE, Na, K, AST, and UN provides a reliable estimation of corresponding serum values in pigs, representing a useful diagnostic and forensic tool in the case of animal welfare.

## Simple Summary

This study evaluates whether aqueous humor can serve as a reliable substitute for blood in postmortem biochemistry diagnostics in pigs. Blood becomes unsuitable after death due to rapid decomposition, while eye fluid remains stable and protected. Samples from healthy pigs showed strong correlations between aqueous humor and serum values for urea, creatinine, sodium, potassium, and phosphorus, allowing estimation of antemortem conditions. In a case of ventilation failure causing death in pigs, eye fluid analysis revealed biochemical changes consistent with heat stroke and hypoxia. Notably, elevated potassium and creatinine, along with low glucose levels, indicated severe thermal stress and rapid postmortem changes. Sodium levels were less reliable due to temperature effects. Overall, aqueous humor proved to be a stable, practical, and informative matrix for forensic investigations, especially when blood is unavailable, improving diagnostic accuracy and aiding in determining causes of death in pigs.

## 1. Introduction

In diagnostic practice in pigs, using blood biochemical parameters, one of the fundamental diagnostic tools, is often unavailable during necropsy, which can significantly reduce diagnostic accuracy [1,2]. Immediately after death, a series of unavoidable, irreversible, and progressive physical and chemical changes begins, occurring in a defined sequence but at rates that vary considerably depending on environmental conditions [3,4]. Due to rapid autolytic and putrefactive changes in blood, its reliability for biochemical analysis is substantially limited, especially if samples are not collected postmortem [3,5,6]. Forensic biochemistry, or thanatochemistry, is the systematic study of biochemical changes in body fluids after death to provide essential information regarding the cause, manner, and time of death [7,8,9,10]. In cases of animal abuse, cruelty, or violation of welfare laws, this discipline serves as a vital tool for objective assessment by identifying perimortem physiological distress and metabolic imbalances that standard macroscopic examination may fail to detect [7,8,11,12]. The use of eye fluids in postmortem biochemical diagnostics offers significant advantages, as it is anatomically isolated within the eyeball, protecting it from the rapid bacterial contamination and putrefaction processes that affect blood [7,9,10,11,13]. Unlike serum, vitreous humor is almost entirely free of cells, preventing unreliable results caused by postmortem cell degradation, such as hemolysis or bacterial metabolism [13,14]. Many biochemical parameters, including urea and creatinine, remain exceptionally stable in the eye for up to 24 h after death, accurately reflecting the antemortem state of the organism even when blood is biochemically unusable [11,15,16]. Sampling is straightforward, and the clear consistency of aqueous humor often permits direct analysis without centrifugation, facilitating forensic work in the field [13,17]. Although often described as “straightforward” and “easily collected”, proper sampling requires careful technique to preserve sample integrity [18]. To ensure reliable biochemical results, contamination with blood or iris tissue must be avoided, and only clear, colorless fluid should be used for analysis [17,19,20,21]. While changes in postmortem blood are often chaotic, parameters such as potassium in the eye change in a predictable and linear manner, allowing precise estimation of the time since death [10,22,23]. Due to this stability and resistance to decomposition artifacts, eye fluid represents a superior matrix for forensic analysis and the objective assessment of animal welfare immediately prior to death [7]. Studies conducted in humans and various animal species, including horses [24,25], cattle [18,26,27,28,29], sheep [18,28,30], dogs [26,31,32], cats [16,26], rabbits [33], and wildlife [17,20,34], have demonstrated that postmortem aqueous humor can be used to estimate antemortem serum biochemical values [26,27,35]. Nevertheless, despite evidence of correlations between certain analytes in eye fluids and blood, data for pigs remain limited [8,29,36]. In cases of suspected animal abuse, veterinary authorities and the State Attorney’s Office rely on veterinary pathologists to use forensic science methods to objectively determine the cause and circumstances of injury and death, as shown by the case in which pigs died from heatstroke after a suspected mass depopulation event involving a ventilation shutdown [37]. In modern industrial pig production, powerful ventilation systems are a key technological component that enable the maintenance of animal life and productivity under conditions of high stocking density [38]. These systems continuously remove excess body heat, ammonia, and other harmful gases, thereby preventing the development of life-threatening microclimatic conditions within enclosed facilities. However, such reliance on technology also creates significant vulnerability within the production system. Any interruption in ventilation, particularly during periods of high ambient temperatures, can rapidly lead to fatal hyperthermia and respiratory distress in animals [39]. Under such conditions, indoor temperatures may exceed 40 °C, while toxic gases such as ammonia, methane, and hydrogen sulfide accumulate, further worsening the situation [38,40]. Pigs are particularly susceptible to heat stress due to their limited thermoregulatory capacity, making them highly prone to heatstroke and hypoxia [39]. Clinical signs include rapid and labored breathing, open-mouth breathing, muscle tremors, reduced mobility, and, in terminal stages, convulsions and cyanosis [41]. Despite legal requirements mandating the implementation of safety systems such as backup generators, their inefficiency or inadequate maintenance continues to pose a serious risk to animal welfare. Mass depopulation methods based on ventilation shutdown, used in emergency situations, are contrary to European Union regulations, particularly Council Regulation (EC) No 1099/2009 on the protection of animals at the time of killing, and are considered by scientific and regulatory bodies to be highly painful and unacceptable [42]. In this context, the described case of ventilation system failure on a farm, resulting in the death of 56 finishing pigs, further emphasizes the importance of timely and accurate diagnostics, with biochemical analysis of aqueous humor playing a key role in confirming the cause of death.

The present study aimed to investigate whether postmortem concentrations of albumin (ALB), alkaline phosphatase (ALP), alanine aminotransferase (ALT), amylase (AMY), total bilirubin (TBIL), urea nitrogen (UN), creatinine (CRE), calcium (Ca), phosphate (PHOS), sodium (Na), potassium (K), glucose (GLU) and total protein (TP) in aqueous humor reflect serum levels and thus can be used to identify antemortem imbalances and enhance veterinary forensic pathology.

## 2. Materials and Methods

### 2.1. Animals and Sampling

A total of 30 clinically healthy finishing pigs (Landrace breed) were included in the study. They were selected from three commercial farms and slaughtered in an abattoir to establish baseline values and linear regression models (control–baseline group). From each selected pig, blood and aqueous humor samples were collected during exsanguination. Aqueous humor was aspirated from both eyes with a 21-gauge needle inserted horizontally beneath the cornea into the anterior chamber, avoiding the iris tissue [7,11]. The collected samples were pooled and transferred into two plain tubes without anticoagulants and kept on ice. In the laboratory, blood samples were centrifuged at 1600× *g* for 10 min to obtain serum. Aqueous humor samples were centrifuged, and the resulting supernatant was harvested and stored at −20 °C. Following a ventilation system failure on a commercial farm that resulted in the death of 56 finishing pigs, a pathologist randomly selected 15 animals for necropsy and collection of aqueous humor samples according to the same protocol in the decomposed pig carcasses (case–forensic group).

### 2.2. Experimental Design

The experimental design was developed to provide a clear and objective forensic approach to address advanced stages of postmortem decomposition, where classical pathological examination was no longer reliable. A control group of healthy animals was used to establish regression equations linking postmortem aqueous humor concentrations with corresponding antemortem serum values of significantly related biochemical parameters. These equations enabled indirect reconstruction of physiological conditions before death in the forensic (case) group by calculating estimated serum concentrations from postmortem aqueous humor samples. The reconstructed values were then interpreted to identify key forensic indicators, including evidence of physiological distress, differential diagnosis of the cause of death, and assessment of potential intentional animal abuse.

All procedures complied with institutional ethical standards for animal research.

### 2.3. Analytical Procedures

Chemical analyses on aqueous humor and serum included ALT, ALB, ALP, AMY, Ca, CRE, UN, GLOB, GLU, PHOS, K, Na, TBIL, and TP, and were performed using an Abaxis VetScan VS2 (Abaxis, Inc., Union City, CA, USA) chemistry analyzer and the Abaxis Comprehensive Diagnostic Profile reagent rotors (ABAXIS Europe GmbH, Griesheim, Germany). Only colorless and clear aqueous humor was used for analysis [7,24,43,44]. In some samples, viscosity following freezing and centrifugation initially blocked analysis as the machine could not process the sample from the rotor. Such samples were heated at 56 °C for 30 min to reduce viscosity [45], and then centrifuged at 1500× *g* for 10 min.

The VetScan analyzer provides a calculated value for GLOB levels rather than a direct measurement and defaults to reporting a value of 0 when results are <1 g/dL. As all GLOB values in this study were ≤1.0 g/dL and therefore reported as 0 g/dL, calculated GLOB levels, derived by GLOB = TP − ALB as recommended by the Cornell University College of Veterinary Medicine (2022), were used for the precision and stability analyses [46].

### 2.4. Statistical Analysis

The required sample size was determined using a standard *t*-test for comparing two means. To achieve a statistical power of 0.80 with a significance level (alpha) of 0.05 and an expected mean difference of 0.8, a total of 26 participants were required per group. We checked the normality of the distribution of the obtained results using the Shapiro–Wilk test. Correlation coefficients were calculated to assess the association between aqueous humor and serum values. Parameters showing r > 0.6 were subjected to linear regression analysis. Simple linear regression was used to obtain a regression equation for the estimation of the antemortem serum concentration from the postmortem aqueous humor concentration. Statistical significance was set at *p* ≤ 0.05. Data were analyzed using Stata (version 18; StataCorp LLC, College Station, TX, USA).

## 3. Results

**Figure 1:**
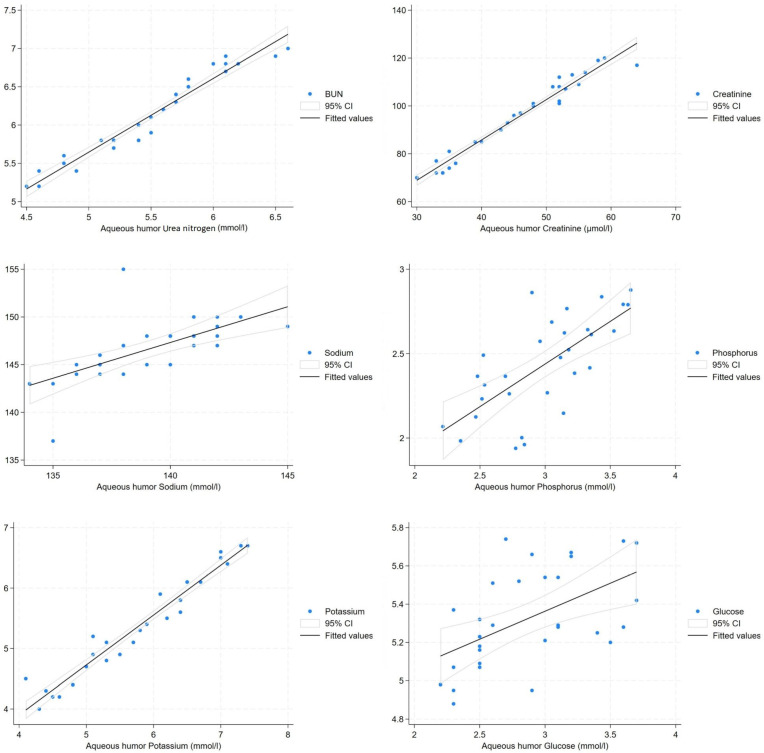
Scatter graph showing fitted regression lines with 95% prediction intervals for pig serum concentrations on aqueous humor concentrations.

Concentrations (mean ± SE) of the various biochemical parameters in serum and aqueous humor are presented in Table 1. The regression of serum urea nitrogen and creatinine on their respective aqueous humor values in pigs indicated a high correlation and a highly significant slope (*p* < 0.001). Significant and strong correlations (*p* = 0.001 and r > 0.6) were also observed for the electrolytes phosphorus, sodium, and potassium. A weak correlation (r = 0.536) was recorded for glucose (*p* = 0.02). Figure 1 shows the regression lines of serum concentrations on aqueous humor concentrations, with a significant relationship (*p* < 0.05).

All autopsied pigs were in an advanced stage of decomposition, consistent with findings indicative of heat stroke and hypoxia following ventilation failure. According to reports from the police and veterinary authorities, the farmer stated the ventilation shutdown was caused by a malfunction in the farm’s electrical system approximately 12 h before the autopsy. Linear regression equations and the extrapolated serum values derived from these models for the examined parameters are presented in Table 2.

## 4. Discussion

Biochemical analysis of aqueous humor in pigs immediately after slaughter provides a robust baseline for interpreting the antemortem physiological status of clinically healthy animals [8,29,30,36,47,48]. In the present study, slaughterhouse samples from 30 finishing pigs demonstrated that concentrations of selected analytes in the aqueous humor can reliably predict their serum counterparts. The strongest correlations were observed for urea nitrogen, creatinine, sodium, and potassium. Notably, the correlation for urea nitrogen in finishing pigs (r = 0.971) exceeded values previously reported by Drolet et al. (r = 0.78 for aqueous humor and 0.69 for vitreous humor) [36], similar to that for cats (r = 0.926) [26], reinforcing the concept that urea nitrogen remains a stable and reliable indicator for at least 24 h postmortem [21,24,26]. Creatinine showed a similarly strong association (r = 0.97), consistent with findings in other species and supporting its use as a proxy for pre-mortem renal function [9,11,13,21,26,30]. Although we observed a moderate correlation (r = 0.702) shortly after death, phosphorus is generally considered a less reliable marker of antemortem status in serum, as its levels in the eye progressively and significantly increase postmortem owing to retinal autolysis [24,26,29,36].

In short postmortem intervals, sodium levels in the aqueous humor generally correspond to those in serum, consistent with our results [11,19,21,29,49]. However, deviations may indicate antemortem electrolyte imbalances, as elevated sodium and chloride levels can result from dehydration, saltwater drowning, or salt intoxication [50,51,52]. In contrast, larger molecules such as amylase, bilirubin, and total proteins exhibited weaker correlations, consistent with the restrictive properties of the blood–ocular barrier [7,26,30,47].

The initial potassium concentrations measured when samples are collected at the moment of death (mean 5.86 mmol/L) closely matched baseline values of approximately 5.5 mmol/L reported in other studies, confirming the stability of this parameter as a reference point for postmortem interval (PMI) estimation in pigs [8,36,48]. The observed postmortem increase in potassium and phosphorus aligns with earlier findings, which documented approximately 1.7- to 2-fold rise in potassium within 24 h [8,36,48], underscoring that electrolyte accumulation due to retinal and, to a lesser extent, lens autolysis is consistent and fairly linear over time, changing at a relatively constant rate [5,10]. Baseline glucose levels in the aqueous humor of healthy pigs were approximately 2.2 mmol/L, but declined postmortem rapidly due to glycolysis. In our study, aqueous humor glucose in slaughtered pigs (2.87 mmol/L) was approximately half of the corresponding serum concentration (5.33 mmol/L), fully consistent with the widely accepted forensic rule that ocular glucose approximates 50% of serum values due to rapid rate of glycolysis [9,11,19,26,53]. Our study showed that estimating antemortem serum calcium values from postmortem aqueous humor appears to be severely limited in swine (r = 0.056), as has been reported in other studies [7,13,30,47]. Collectively, these controlled baseline data provide a critical reference framework for distinguishing physiological from pathological changes in forensic investigations [10,11,13,32].

Under conditions of mass mortality caused by ventilation system failure, the biochemical profile of the aqueous humor shifts markedly due to extreme thermal stress. Analysis of 15 autopsied pigs that died from heat stroke revealed distinct alterations (Table 2) indicative of high-level stress, accelerated postmortem metabolism and severe tissue damage [9,11,13]. Linear regression models for urea nitrogen (y = 0.844 + 0.962x) and creatinine (y = 18.367 + 1.685x) enabled the estimation of expected physiological values, which served as a reference baseline for immediate postmortem conditions. For instance, in slaughtered pigs, mean aqueous humor glucose was 2.87 mmol/L, while phosphorus remained stable at approximately 2.99 mmol/L.

In contrast to these expected values, the biochemical findings in pigs that died from heat stroke demonstrated profound pathological deviations. The most striking change was observed in potassium levels, which increased from an expected baseline of approximately 5.86 mmol/L to a mean of 12.33 mmol/L. Although postmortem potassium elevation is a well-established phenomenon, such markedly elevated levels within a relatively short PMI strongly indicate accelerated retinal autolysis driven by extreme hyperthermia [7,8,24,29]. Glucose dynamics further support this interpretation: the markedly reduced concentration of 1.3 mmol/L reflects both intense perimortem energy depletion and rapid postmortem anaerobic glycolysis, characteristic of severe thermal stress [7,8,9,26,40]. As often occurs, heat stroke is accompanied by severe dehydration; initial sodium concentrations in eye fluids may be markedly elevated and often exceed 150 mmol/L and, in extreme cases, reach above 200 mmol/L [7,9,10,11,40]. However, due to the accelerated postmortem decline driven by elevated temperatures, heat can effectively “mask” antemortem dehydration by rapidly lowering sodium concentrations to seemingly normal values [7,9,10,19,24,29,43]. Therefore, in such cases, sodium levels are considered unreliable for diagnosis of dehydration [9,51]. In this case, one of the questions for the forensic pathologist was as follows: Was the ventilation shutdown intentional or accidental? Increased sodium levels could indicate severe dehydration in animals following water deprivation and could change the classification of the act towards intentional and planned animal killing.

Similarly, elevated phosphorus (5.38 mmol/L), along with increased amylase (58.47 U/L) and alkaline phosphatase (35.73 U/L), indicates substantial leakage of intracellular components due to thermally induced membrane disruption and ocular tissue autolysis [7,26,36]. From a forensic perspective, the most diagnostically informative finding is the relationship between creatinine and urea nitrogen [7,9,11,13]. The observed isolated creatinine elevation of concentration (mean 149.67 μmol/L), in conjunction with a relatively stable urea nitrogen (5.55 mmol/L), constitutes a distinctive biochemical “signature” of fatal heat stroke [11,13]. In contrast to renal failure, which would typically produce a parallel increase in both markers, the isolated elevation of creatinine in the early postmortem period strongly supports death due to acute thermal overload and is not consistent with prolonged dehydration, supporting the forensic conclusion that the ventilation shutdown was likely unintentional [7,9,11,26,51].

### Study Limitations

Despite the considerable diagnostic value of aqueous humor analysis, several limitations of this study should be acknowledged. The sample size, although adequate for establishing initial correlations, should be increased in future studies to include a wider range of stress conditions and production systems to improve general applicability. Methodological aspects, particularly the aspiration technique, require strict quality control and training to prevent contamination that may affect biochemical results. In addition, the intrinsic properties of aqueous humor, such as increased viscosity due to hyaluronic acid, present analytical challenges, while the limited sample volume restricts the number of parameters that can be assessed. It should be noted that the lack of standardized pre-analytical protocols may limit comparability with findings from other studies.

## 5. Conclusions

Postmortem aqueous humor analysis in pigs provides a reliable means for estimating antemortem serum biochemical concentrations of urea nitrogen, creatinine, sodium, potassium, phosphorus and, to a lesser degree, glucose. The integration of regression-derived baseline values with empirical findings from autopsied animals enables a robust and objective forensic framework for confirming heat stroke as the cause of death. These results provide compelling biochemical evidence of extreme physiological distress in affected animals and highlight the diagnostic value of aqueous humor analysis in large-scale mortality events. Given that species-specific ocular biochemical data in pigs have historically been sparse and often outdated, and frequently exceed three decades in age, this study establishes an essential contemporary reference for the accurate interpretation of pathological deviations in cases involving compromised animal welfare due to hyperthermia.

Although sampling in our study was performed with care, the possibility of blood contamination during puncture cannot be entirely excluded. Therefore, assessment of the hemolysis index is recommended to confirm that samples are free from blood. Moreover, electrolyte levels may vary between ocular compartments [34], potentially introducing bias into the study.

These findings support the use of aqueous humor as a diagnostic substitute for blood samples in forensic veterinary pathology.

## Figures and Tables

**Table 1:** Biochemical analysis results of serum and aqueous humor in control (baseline) pigs after slaughter.

Biochemical Parameter	Serum ^1^		Aqueous Humor ^1^		*R* ^2^	Linear Regression Equation	*p*-Value
	Mean ± SE	Min–Max	Mean ± SE	Min–Max			
ALB (g/L)	31.4 ± 1.26	18–48	0.57 ± 0.02	0.4–0.8	0.191		0.307
ALP (U/L)	203.4 ± 10.33	117–295	16.87 ± 0.81	8–25	0.099		0.601
ALT (U/L)	45.83 ± 1.47	28–59	5.17 ± 0.52	0–12	−0.012		0.949
TBIL (mmol/L)	7.33 ± 0.91	1–24	0.23 ± 0.01	0.2–0.3	0.212		0.259
UN (mmol/L)	6.15 ± 0.1	5.2–7	5.52 ± 0.1	4.5–6.6	0.971	y = 0.844 + 0.962x	0.001
Ca (mmol/L)	2.49 ± 0.13	1–4	1.8 ± 0.07	1.1–2.5	0.056		0.768
PHOS (mmol/L)	2.43 ± 0.05	1.94–2.88	2.99 ± 0.07	2.21–3.66	0.702	y = 0.924 + 0.505x	0.001
CRE (µmol/L)	94.7 ± 3.01	70–120	45.3 ± 1.75	30–64	0.981	y = 18.367 + 1.685x	0.001
GLU (mmol/L)	5.33 ± 0.05	4.88–5.74	2.87 ± 0.08	2.2–3.7	0.535	y = 4.484 + 0.293x	0.002
Na (mmol/L)	146.7 ± 0.57	137–155	139.2 ± 0.49	134–145	0.642	y = 42.409 + 0.749x	0.001
K (mmol/L)	5.44 ± 0.15	4–6.7	5.86 ± 0.18	4.1–7.4	0.973	y = 0.597 + 0.826x	0.001
TP (U/L)	62.9 ± 1.77	52–89	1.37 ± 0.04	1–1.8	0.017		0.929
GLOB (U/L)	52.7 ± 1.91	28–72	0.8 ± 0.03	0.5–1	0.027		0.882
AMY (U/L)	1428.53 ± 41.66	1005–1825	37.23 ± 6.32	7–128	0.036		0.849

^1^ Data are presented as mean ± standard error (SE) for 30 clinically healthy finisher pigs (*n* = 30) selected from three commercial farms. Abbreviations: ALB—albumin; ALP—alkaline phosphatase; ALT—alanine aminotransferase; TBIL—total bilirubin; UN—urea nitrogen; Ca—calcium; PHOS—phosphate; CRE—creatinine; GLU—glucose; Na—sodium; K—potassium; TP—total protein; GLOB—globulins; AMY—amylase. Technical notes: Calculated GLOB levels (GLOB = TP − ALB) were used because the analyzer defaults to 0 when results are <1 g/dL. *R*^2^ indicates the coefficient of determination for the correlation between serum and aqueous humor; *p*-value represents statistical significance (*p* ≤ 0.05). The linear regression equation is provided for parameters with a correlation coefficient r > 0.6.

**Table 2:** Biochemical analysis of aqueous humor and corresponding serum values estimated using linear regression models in forensic case pigs.

Biochemical Parameter	Mean ± SE ^1^	Min–Max	Extrapolated Value from Linear Regression Equation *
ALB (g/L)	0.80 ± 0.04	0.5–1	
ALP (U/L)	35.73 ± 1.51	28–48	
ALT (U/L)	4.93 ± 0.86	0–12	
TBIL (mmol/L)	0.23 ± 0.01	0.2–0.3	
UN (mmol/L)	5.55 ± 0.17	4.5–7.1	6.183
Ca (mmol/L)	1.79 ± 0.09	1.2–2.2	
PHOS (mmol/L)	5.38 ± 0.15	4.25–6.68	3.641
CRE (µmol/L)	149.67 ± 2.5	135–168	270.561
GLU (mmol/L)	1.30 ± 0.00	1.3–1.3	4.685
Na (mmol/L)	137.47 ± 2.28	121–155	145.374
K (mmol/L)	12.33 ± 0.34	10.2–14.5	10.782
TP (U/L)	1.77 ± 0.09	1.3–2.4	
GLOB (U/L)	0.75 ± 0.03	0.5–0.9	
AMY (U/L)	58.47 ± 5.29	30–90	

^1^ Data are presented as mean ± standard error (SE) for 15 finishing pigs (*n* = 15) randomly selected from a population of 56 animals that died following a ventilation system failure on a commercial farm. * Extrapolated value from linear regression equation: These values (x = estimated mean value) represent the estimated antemortem serum concentrations, derived by applying the linear regression equations (e.g., y = 0.844 + 0.962x for UN) established from the control group of 30 healthy pigs to the postmortem aqueous humor results of the deceased animals.

## Data Availability

The data presented in this study are available on request from the first author.
